# Nicaragua's surprising response to COVID-19

**DOI:** 10.7189/jogh.10.010371

**Published:** 2020-06

**Authors:** Andy A Pearson, Andrea M Prado, Forrest D Colburn

**Affiliations:** 1 INCAE Business School, Alajuela, Costa Rica; 2 The City University of New York, New York, New York, USA

Nicaragua’s response to COVID-19 has been surprising; the government has not only dismissed the recommendations of world health authorities to take precautions but has flouted them by organizing large-scale public events [1]. The Nicaraguan government’s dismissal of the danger of COVID-19 has not only led to an international outcry, especially from neighboring countries, but has also put Nicaraguan doctors and other health professionals in an agonizing dilemma. While nominally a democracy, Nicaragua has come to be governed by an all-powerful husband and wife: the government of President Daniel Ortega and his wife, Vice President Rosario Murillo [2]. They together govern as a dictatorship, with no institutional check on their authority. They are subject to no public accountability or even pressure exerted by interest groups.

Consequently, Ortega and Murillo can be – and are – capricious. Managua’s streets, for example, are adorned with two-hundred or so garish, metal “Trees of Life,” 42 to 56 feet high, inspired by a Gustav Klimt painting. They glow at night thanks to some 2.5 million small electric bulbs (in a country where many homes lack electricity). Murillo decided Nicaraguans needed inspiration. Neither Congress nor any other representative body had a say in erecting the trees. Health policy during a pandemic is a severe issue. The case of Nicaragua, though, suggests that even in the time of a crisis threatening the lives of an entire nation, leaders of authoritarian governments, free from any kind of public censure, are likely to be more concerned with preserving their rule – or even their image – than with protecting citizens from a public-health disaster [3].

Moreover, authoritarian governments, such as that of Ortega and Murillo, may be powerful enough to turn professional bureaucracies, such as those of health, into sycophants. The COVID-19 threat in Nicaragua is worrisome not only because government policy is to dismiss the threat, but also because the country’s health care professionals have been cowered by threats, and a generalized atmosphere of fear, not to challenge reckless government policy. Nicaraguan health care professionals protest, even when told not to wear masks while working, at great professional and even personal risk. The COVID-19 crisis in Nicaragua is thus truly alarming. Nicaraguans, including prominently doctors and nurses, have been left to the uncertain vicissitudes of the novel coronavirus.

Nicaragua is the second poorest country in the Western Hemisphere. In 2018, the country’s gross domestic product (GDP) per capita was US$ 1860 (compared to an average for US$ 9889 for Latin American and Caribbean countries), and 50.5 percent of its population was living under the poverty line. For the same year, its population was approximately 6.5 million people, with 41.5 percent living in rural areas. Managua, the nation’s capital, housed one-fourth of Nicaragua’s population. According to the 2019 Social Progress Index (based on multiple indicators showing several social metrics, such as health care and basic medical attention), Nicaragua ranked 103th among 149 countries, with a score of 58.97/100. Government health expenditure in 2017 was five percent of its GDP, distributed among three public institutions: the Ministry of Health, the Nicaraguan Social Security Institute, and the Defense Ministry. In terms of infrastructure to face this pandemic, Nicaragua has 0.9 hospital beds per 1000 inhabitants (compared to 2.2 for Latin America and the Caribbean at large) and an estimated stock of 160 ventilators, of which 80 percent are currently in use [4]. Given the country’s poverty, scarce resources must be judiciously employed – and so government policies are all-important.

As of May 5, the Nicaraguan Ministry of Health reported 15 COVID-19 confirmed cases, 16 suspected cases, and five deaths. Nevertheless, it is not clear how many tests per day have carried out since the first case was reported on March 19. The Nicaraguan Health Ministry forecasts 32 500 COVID-19 cases, of which 25 percent would be severe or critical, and that 813 people would die between March and August 2020 [5]. According to the Ministry of Health’s epidemiological bulletin, acute respiratory infections and pneumonia cases for week 18 of 2020 decreased 15.6 percent and 20.7 percent, respectively, compared to the same week of 2019. Pneumonia deaths also decreased from 146 in 2019 to 86 in 2020 [6]. Thus, the situation publicly appeared to be under control by May 7, even though, as different countries around the world have seen, the COVID-19 case curve may grow exponentially at any moment.

However, different groups are raising questions regarding official reports on COVID-19 cases in Nicaragua. Given the opacity of the official data, the Citizen Observatory for COVID-19, a multidisciplinary group of self-organized Nicaraguans, monitors suspected cases and registers irregularities in the health system based on citizen reports. The group makes an unofficial and unconfirmed count of the suspected cases of COVID-19. As of May 4, they recorded 601 suspected cases of COVID-19 across the country [7]. The Nicaraguan press reported almost 90 COVID-19 patients, 14 of those cases requiring a ventilator, in the most critical public hospital of Nicaragua. Also, the Nicaraguan Medical Union stated that in the country, there are more than 40 cases of COVID-19 infections in medical personnel.

Moreover, the government has restricted actions by nongovernmental and religious groups to provide health support to the population. For instance, the Diocese of Matagalpa was not allowed to operate a call center and provide free COVID-19 related advice to community members. Restrictions to civil society institutions limit the ability to identify potential COVID-19 cases and to report to national and international observers. The motivation for controlling information is perceived to be for political reasons. As one Nicaraguan doctor says, “When the government controls information about the virus, it does so to calm fears among Nicaraguans that they are at risk” (personal communication, April 29, 2020). The doctor reports, too, that he is constrained: “If I want to keep my position [as a doctor], I must do what the government says.” Another doctor, one working in a rural part of the country, suggests that in the countryside there are even more constraints for health professionals than those faced in urban areas; she reports, “If I tell someone they should stay indoors I am considered to be part of the political opposition” (personal communication, April 29, 2020). She and other health professionals, feel intimidated. There is no public discussion in Nicaragua of how the country should respond to the pandemic; the country’s response is decided by Ortega and Murillo, and seemingly based on what serves their desire to retain absolute political control of the country.

## REAPPEARING TO REWRITE HISTORY

President Ortega disappeared from public sight for 34 consecutive days once the COVID-19 crisis started in Nicaragua, leaving Vice President Murillo in command. He reappeared on April 15, delivering a broadcasted speech from a room with ten cabinet members, including the Vice President and the Minister of Health [8]. Ortega stated that Nicaragua was complying with the measures that the international health organizations recommended for coping with COVID-19. He claimed that health professionals went house-to-house, educating families on how to protect themselves from the virus. Ortega congratulated different public institutions (eg, Ministry of Health, police, and military forces) for holding back the spread of the COVID-19 cases in the country. Nicaragua was, he asserted, the country in Central America with the lowest number of COVID-19 cases.

**Figure:**
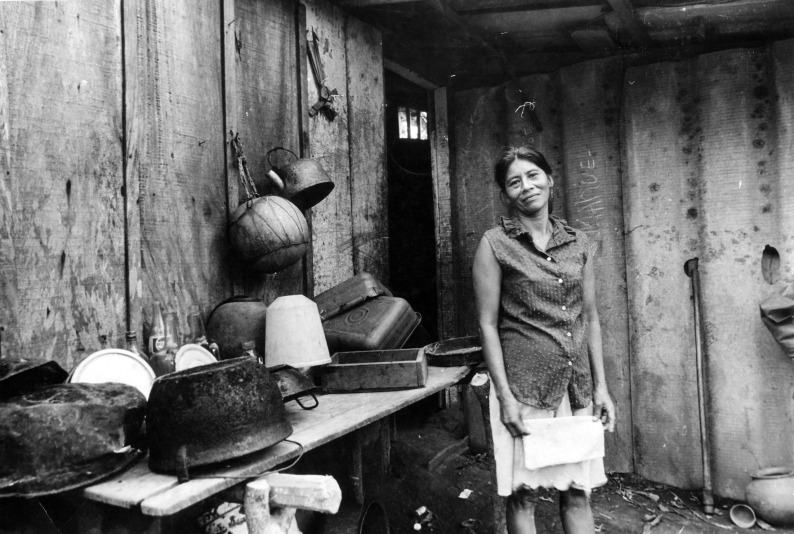
Photo: Nicaragua is the second poorest country in the Americas, posing a challenge to health care. (Photograph by Celeste González, Haciendo la Reforma, Carazo, Nicaragua, 1984; used with permission).

Ortega addressed the economic dimension of the crisis in his speech and used it to explain why the country is not in lockdown. Nicaragua has been in a political and economic crisis since April 2018, when the government proposed reforms of the pension system and citizens rebelled, first against the reforms of the pensions and then against continued rule by Ortega and Murillo. In addition to multiple human rights violations reported in Nicaragua since the conflict started, the country’s economy showed a 4.0 and 3.9 percent decrease in its GDP in 2018 and 2019, respectively [9]. Ortega justified the absence of quarantine by publicly stating, “We have not stopped working because if we stop working, the country dies, and if the country dies, the people die” [8]. According to the World Bank’s revised economic forecast for 2020 – including the COVID-19 effects – Nicaragua will face its third consecutive year of economic recession with a 4.3 percent decrease in its GDP, badly hurting an economy that is already significantly weak. Ortega and Murillo understand that economic contractions easily lead to demands for political change.

In a bid to deflect attention to Nicaragua, President Ortega criticized how wealthy countries and multilateral organizations have dealt with the pandemic. He asserted that God was sending a message to developed countries – like the United States – to invest more in hospitals and health centers, rather than in arms and bombs. He also demanded the ethical and moral renovation of international organizations like the United Nations [8]. The only reference regarding neighboring countries was an incident regarding a pregnant Nicaraguan teenager who illegally crossed the border into Costa Rica (she was tested for COVID-19, but the results were negative). Nevertheless, the health and economic crises that one might anticipate in Nicaragua are likely to increase migration to other Central American countries. This migration, though, is not a political threat to Ortega and Murillo.

## BEYOND NICARAGUAN BORDERS: COMPARISONS AND SPILLOVER EFFECTS

Most countries around the world are following the recommendations of international health organizations, but there are exceptions. As in Nicaragua, Sweden has followed a controversial strategy to face COVID-19. Comparing their approach, both countries decided to keep their economies open, but in the case of Sweden, the government closed all activities related to the educational sector and banned large gatherings. The Nicaraguan government, instead, kept schools and universities open – only closing them for two weeks due to Easter celebrations – and promoted cultural events across the country. Just as importantly, Sweden has an explicit health policy – herd immunity. Sweden’s ambassador to the US has stated as much in an interview given on April 26: “The country's controversial strategy of pursuing ‘herd immunity’ – and not locking down the country – is bearing success, with the capital Stockholm on course to reach herd immunity in the next few weeks” [10]. Nothing Ortega or Murillo have said, though, suggests that they are shaping Nicaragua’s response to the pandemic based on a desire to build herd immunity. Instead, their public utterances suggest the same determination to maintain political support for their regime as led Ortega and Murillo to repress savagely protests against their rule in 2018 and 2019, even to the point of using snipers to assassinate unarmed student protestors [11].

Moreover, Sweden and Nicaragua are unevenly equipped to face the outcomes of forgoing a quarantine. For example, the Social Progress Index ranks Sweden in the 11^th^ place in Health and Wellness, and Nicaragua in the 47^th^ place. According to the World Bank database, the number of hospital beds per 1000 people in 2013 was 2.6 and 0.9 for Sweden and Nicaragua, respectively [12]. In March 2020, the Nicaraguan Ministry of Health reported that 17 health care centers around the country were equipped to treat COVID-19 patients but gave no details on *how* they would be treated [5]. Doctors – and hospitals – are better prepared in Sweden, one of the wealthiest countries in the world.

Confirmed COVID-19 cases and deaths data from Sweden and Nicaragua also differ. As of May 7, 2020, Sweden is experiencing higher numbers of COVID-19 cases (24 623) and deaths (3040) in comparison with neighboring countries such as Norway (8015 cases and 217 deaths) and Denmark (10 083 cases and 514 deaths) [13]. The difference in the number of cases could be expected, considering that the population in Sweden (10.2 million) almost doubles that one of Norway (5.3 million) and Denmark (5.8 million). However, the number of deaths shows a disproportionate difference with mortality rates of 12.3, 2.7, and 5.1 percent for Sweden, Norway, and Denmark, respectively. Nicaragua, on the other hand, is reporting lower numbers of COVID-19 cases (15) and deaths (five) in comparison with neighboring countries such as Costa Rica (765 cases and six deaths) and Honduras (1461 cases and 99 deaths). Population sizes and mortality rates also vary within these three countries. With populations of 6.5 million in Nicaragua, 5.0 in Costa Rica, and 9.6 in Honduras, the corresponding mortality rates are 33.3, 0.78, and 6.77 percent. It is clear from these figures that Nicaragua has a disproportionately high mortality rate (according to official statistics). The fear, including among Nicaraguan health professionals, is 2-fold: 1) that official statistics are grossly manipulated for political reasons, and 2) that mortality might sharply increase because Ortega and Murillo refuse to recognize the seriousness of the crisis.

Nicaragua’s COVID-19 strategy is likely to have health and social consequences for neighboring countries, particularly for Costa Rica, which traditionally receives a high annual influx of economic migrants from Nicaragua. For instance, during the 2018 Nicaraguan political crisis, Costa Rica received 23 138 asylum applications, compared to 67 requests in 2017 [14]. According to the 2011 Costa Rican Census, 287 766 Nicaraguan immigrants were residing in the country, representing 76.4 percent of the total of international inhabitants [15]. Thus, whatever happens in Nicaragua prompts migration to Costa Rica. Knowledge among Nicaraguans that Costa Rica has one of the most robust health care systems in the Americas is likely to contribute further to migration stimulated by the pandemic.

While Nicaragua is all but ignoring the pandemic, Costa Rica is promoting social distancing and limiting activity in non-essential economic sectors such as tourism, culture, and education. Costa Rica’s president, Carlos Alvarado, is taking the COVID-19 threat seriously. The stark difference between the approaches to COVID-19 between Costa Rica and Nicaragua led Costa Rican authorities to strengthen the vigilance at the northern border, anticipating an increase in the number of illegal immigrants. On March 19, Costa Rica closed all the borders for foreigners (the first COVID-19 case in Costa Rica was confirmed on March 6). By April 12, Costa Rican police patrols had stopped at least 5000 immigrants at the border. Still, Costa Rica installed a field hospital at the border to expand its capacity to serve Nicaraguans, who might cross the border and require health services.

Nicaragua’s dismissive strategy towards COVID-19 is also worrisome to El Salvador and Honduras. Nicaragua’s northern neighbors, El Salvador and Honduras are worried about Nicaragua’s disregard for the threat posed by the pandemic, concerned that sooner or later, the highly infectious disease will be spread by Nicaraguans crossing their porous borders. In both El Salvador and Honduras, borders have been closed, and the population has been directed to quarantine itself. These two developing countries thus have followed, in contrast to Nicaragua, the recommendations of the Pan American Health Organization (PAHO). Besides, in both countries, the medical profession has a voice in shaping public policies for addressing COVID-19. Still, there are worries that the presidents of both countries seek to exploit the crisis for political gain. A doctor in El Salvador states, “President Nayib Bukele is utilizing the COVID-19 crisis to increase his power and to be more authoritarian in running the country” (personal communication, April 28, 2020). The president of Honduras, Juan Orlando Hernández, is unpopular and is judged to be using curfews to quell public protests. A doctor in Honduras worries, too, that even in an emergency threatening public health, essential medical supplies can be fodder for corruption (personal communication, April 30, 2020). Politics is not everything in the developing countries of Central America, but it never seems very far from the surface. A simple comparison of four of the five countries of Central America (leaving out Guatemala), suggests the better the country is governed, the more rational, and coherent, is its response to the COVID-19 threat, with Costa Rica being at one end of the spectrum, and Nicaragua at the other end of the spectrum (with El Salvador and Honduras in the middle). This outcome is not surprising, but economic development in the region overlaps this political spectrum: Nicaragua is the poorest country in Central America and so the least able to afford an irrational strategy for managing the COVID-19.

The small countries of Central America have fragile economies. With the notable exception of Costa Rica, countries are weak, and even in the best of times, struggle to provide adequate health care. The poorest country in the Central American region is Nicaragua, and political conflict has led to economic contractions for the past two and a half years. The COVID-19 is an inopportune crisis. However, the embattled regime of Ortega and his wife Murillo are ignoring it, pretending that it does not exist. A cynical view, but one entertained by health professionals in Nicaragua, is that government restrictions on economic activity would lead to Ortega and Murillo being blamed for hardship, but that the slow, steady death of individual Nicaraguans, here and there throughout the country, would just be perceived by Nicaraguans as the course of nature. It is a cruel political calculus, with untold risks.
